# Presentation intervals and the impact of delay on breast cancer progression in a black African population

**DOI:** 10.1186/s12889-020-09074-w

**Published:** 2020-06-19

**Authors:** Olayide Agodirin, Samuel Olatoke, Ganiyu Rahman, Julius Olaogun, Olalekan Olasehinde, Aba Katung, Oladapo Kolawole, Omobolaji Ayandipo, Amarachukwu Etonyeaku, Olufemi Habeeb, Ademola Adeyeye, John Agboola, Halimat Akande, Soliu Oguntola, Olusola Akanbi, Oluwafemi Fatudimu

**Affiliations:** 1 Department of Surgery, University of Ilorin and University of Ilorin Teaching Hospital, Ilorin, Kwara state Nigeria; 2 Department of Surgery, University of Cape Coast and Cape Coast Teaching Hospital, Cape Coast, Ghana; 3 Department of Surgery, Ekiti State Teaching Hospital, Ado-Ekiti, Ekiti state Nigeria; 4 Department of Surgery, Obafemi Awolowo Teaching Hospital, Ile-Ife, Osun state Nigeria; 5 Department of Surgery, Federal Medical Center, Owo, Ondo State Nigeria; 6 Department of Surgery, LAUTECH Teaching Hospital, Osogbo, Osun state Nigeria; 7 Department of Surgery, University College Hospital, Ibadan, Oyo state Nigeria; 8 Department of Surgery, Obafemi Awolowo Teaching Hospital, Ilesha, Osun state Nigeria; 9 Department of Surgery, University of Ilorin Teaching Hospital, Ilorin, Kwara state Nigeria; 10 Department of Surgery, General Hospital Ilorin, Ilorin, Kwara state Nigeria; 11 Department of Radiology, University of Ilorin and University of Ilorin Teaching Hospital, Ilorin, Kwara state Nigeria; 12 Department of Surgery, LAUTECH Teaching Hospital, Ogbomoso, Oyo State Nigeria; 13 Department of Surgery, Federal Teaching Hospital, Ido-Ekiti, Ekiti state Nigeria

**Keywords:** Breast cancer, Help-seeking, Primary-care, Intervals, Tumor progression

## Abstract

**Background:**

The help-seeking interval and primary-care interval are points of delays in breast cancer presentation. To inform future intervention targeting early diagnosis of breast cancer, we described the contribution of each interval to the delay and the impact of delay on tumor progression.

**Method:**

We conducted a multicentered survey from June 2017 to May 2018 hypothesizing that most patients visited the first healthcare provider within 60 days of tumor detection. Inferential statistics were by t-test, chi-square test, and Wilcoxon-Signed Rank test at *p*-value 0.05 or 95% confidence limits. Time-to-event was by survival method. Multivariate analysis was by logistic regression.

**Results:**

Respondents were females between 24 and 95 years (*n* = 420). Most respondents visited FHP within 60 days of detecting symptoms (230 (60, 95% CI 53–63). Most had long primary-care (237 of 377 (64 95% CI 59–68) and detection-to-specialist (293 (73% (95% CI 68–77)) intervals. The primary care interval (median 106 days, IQR 13–337) was longer than the help-seeking interval (median 42 days, IQR 7–150) Wilcoxon signed-rank test *p* = 0.001. There was a strong correlation between the length of primary care interval and the detection-to-specialist interval (r = 0.9, 95% CI 0.88–0.92). Patronizing the hospital, receiving the correct advice, and having a big tumor (> 5 cm) were associated with short intervals.

Tumors were detected early, but most became advanced before arriving at the specialist clinic. The difference in tumor size between detection and arriving at a specialist clinic was 5.0 ± 4.9 cm (95% CI 4.0–5.0). The hazard of progressing from early to locally advanced disease was least in the first 30 days (3%). The hazard was 31% in 90 days.

**Conclusion:**

Most respondents presented early to the first healthcare provider, but most arrived late at a specialist clinic. The primary care interval was longer than the help-seeking interval. Most tumors were early at detection but locally advanced before arriving in a specialist clinic. Interventions aiming to shorten the primary care interval will have the most impact on time to breast cancer presentation for specialist oncology care in Nigeria.

## Background

Breast cancer (BC) patients in low and middle-income countries (LMICs) and black patients in developed countries harbor symptoms for up to 8–12 months [1–5] before diagnosis and treatment thereby increasing the risk of poor outcome and limiting treatment efficacy [6–9].

Historically, two delay components are recognized in cancer treatment: the patients’ delay and the systems’ delay [10, 11]. A recent definition proposes replacing the word delay with the word interval [12]. As illustrated in Olsen et al., the Aarhus statement [13] recognized three subintervals between symptom detection and cancer treatment: (1) The patient-interval (comprising symptom appraisal and help-seeking intervals (HSI) as in Dobson et al. [12]) (2). the doctor interval, and (3) the system interval [13]. The subinterval classification aids the understanding of the continuum by giving more details about the subcomponents [12]. Shortening the interval to treatment is pivotal in controlling BC outcome [10], yet in LMICs where the mortality of the disease is disproportionately high, only a few studies address the intervals or journey of BC patients to treatment [14–16].

Factors linked to delayed presentation of breast cancer are often modifiable—changing with intervention. While much of the focus has been on the events in the patient-interval as causes of delayed presentation, recent reports in Nigeria [17], Ghana [18], and Rwanda [3] show an increasing contribution of events in the provider interval. An understanding of factors influencing the length of each interval is critical to effective interventions; therefore, this research aimed to describe the journey of BC patients from symptom detection to the specialist clinic in a black African population. The primary objective was to describe the contribution of each interval to the continuum. The secondary objectives were (1) to describe the association between the interval length and the socio-demographics, the disease-related experience(s), and system-related experience(s) (2) To describe the impact of long intervals on BC progression.

## Method

This research was a questionnaire-based survey in 6 tertiary hospitals in Northcentral and Southwestern Nigeria. The hospitals received referrals from lower cadre public hospitals, private hospitals, or walk-in (self-referral). Recruitment of respondents was between June 2017 and May 2018 after obtaining ethical approval from all participating institutions. Consecutive newly diagnosed BC patients who consented to participate in the study were recruited until the predetermined sample size. At the time of the survey, BC patients in Nigeria patronized private and public healthcare services including traditionalists, native healers, faith-based homes, and orthodox medical healthcare providers (community health extension workers (CHEW), nurses, chemists, pharmacists, and doctors).

Based on piloting on 30 respondents where 80% visited the first healthcare provider (FHP) within 30 days of symptom detection, we hypothesized that most patients would visit their FHP within 60 days. Our sample size was 384, calculating for a descriptive cross-sectional study at a relative precision of 5% and a confidence level of 95% (1.96). We increased the sample size to 423 in anticipation of a 10% nonresponse rate.

### Data collection

Based on insight from the Aarhus statement [13], and review of the methods implemented by Varella-Centelles et al. [19] and Moodley et al. [16], a semi-structured questionnaire was designed and the plan of data collection was mapped. The questionnaire was pilot tested before trained personnel administered it in face-to-face interviews. The questionnaire requested information on socio-demographics, recall of first breast bodily change and events surrounding it, disclosure, and help-seeking patterns (see Additional file 1). The questionnaire was administered to respondents within four weeks of arriving in the specialist clinic (SC) to minimize recall bias. Additionally, respondents were helped to cast their minds back on significant personal, social, religious, regional, or national events surrounding the recalled dates or elapsed periods. Attempts to check for reliability of response or to reconcile discrepancies was by triangulation when possible. [Such as BC awareness, path-way to treatment, personnel(s) visited, tumor size and interval lengths]. Questions likely to influence subsequent response questions were delayed. For instance, religious affiliations were delayed until after responding to the use of alternative medicine. The interview was after the day’s medical consultation in the patient’s mother tongue or English as preferred by the respondent, noting the use of a translator. Recurrent lesions, language barriers, mental incapacitation, and male sex were exclusion criteria.

Clinical tumor size (T-size) estimated by the patient was used as the surrogate for disease stage using the T1–3 as in the 7th edition of the American Joint Committee on Cancer (AJCC) staging for BC, where T1 was ≤2 cm, T2 was 2.1–5 cm, and T3 was > 5 cm. Using the practical routine process of extracting clinical history that relies on the patients’ retrospective recall, respondents were asked to estimate their tumor size (self-report) at the following three points: first detection, first contact with FHP, and at first SC attendance. A ruler was used to quantify the estimated tumor size in centimeters, as demonstrated by the patient using their phalanx, finger(s), or clenched fist(s). The T-size estimated at the SC was taken as the current tumor size. The patients’ estimate was considered unreliable and excluded from analysis if the current size estimated differed by more than 2 cm from the T-size measured physically and recorded by the clinician in the SC.

In defining interval lengths, we used logical arithmetic derivatives of interval lengths in previous works and recommendations [4, 12, 13] wherein total delay was > 90 days, and provider delay was > 30 days. However, to be pragmatic, we considered our health-system and our patients’ behavior to operationalize the interval lengths. Therefore we operationalized the interval lengths as follows:

Appraisal interval (API)—the period from the detection of first breast symptom to first disclosure. Long was > 30 days) (the API was essential to estimate how long the patients keep the lesion secret).

Help-seeking interval (HSI)—the period from symptom detection to FHP. Long was > 60 days)(HSI was essential to estimate how long the patient stayed before seeking help).

**Fig. 1:**
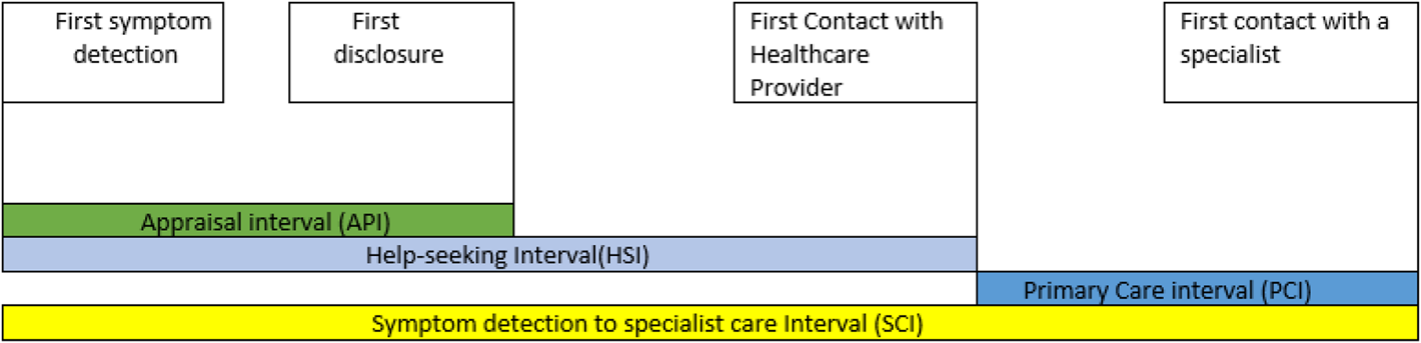
Description of the intervals

Primary-care interval (PCI)— the period from the FHP to a specialist clinic. Long was > 30 days) (PCI was essential to estimate interval length attributable to provider system). And symptom detection-to-specialist clinic interval(SCI)—the period from detection of the first symptom to arriving in a specialist clinic. Long was > 90 days. (SCI was essential because, in Nigeria, the majority of patients receive tumor-specific therapy in specialist clinics, and long SCI is rife) (Fig. 1). We recorded the intervals in days, weeks, or months, multiplying recording made in weeks by 7 to convert to days, and those made in months by 30 to convert to days.

### Statistical analysis

We compared variables using the chi-square test, paired t-test, Wilcoxon Signed-Rank test, and logistic regression for odds of events as appropriate. We used the correlation coefficient for the relationship between continuous variables. We conducted the time-to-event analysis using the survival method on the assumption that tumor progression depended on elapsed time alone. The analysis of progression was limited to 365 days in 30-day time segments because most patients in Africa present within 8–12 months. A statistically significant *p*-value (two-sided) was 5%.

We presented the number of respondents traversing each interval and compared their relative probabilities because we expect that future interventions will focus on increasing the probability of favorable events such as short intervals. We presented a wide range of result patterns because of the dearth of information despite the importance of BC in Africa also because there is a lack of consensus on whether to analyze as parametric or nonparametric variables [12]. Additionally, we expected that these figures might serve different purposes in future researches.

## Results

### Demographics and premorbid preferences

There were 423 respondents; we excluded three males leaving 420 females. The majority were the Yoruba tribe, only one required interpreter. The modal age decade was the fifth (Table 1). Most respondents (323 of 358(90, 95%CI 87–93) [62 unspecified] preferred orthodox medical care before noticing their symptoms, but most did not utilize BC screening. Only 6.0%(95% CI 4.0–9.0%) performed self-breast examination monthly (Table 1b) hence most lumps were detected inadvertently.

**Table 1:** The Demographic characteristics of respondents showing age distribution, educational status, marital status, religion, occupation, tribe, place of the interview, the respondents’ premorbid pattern of help-seeking for medical service and the premorbid utilization of breast cancer screening modalities

a. Demographic characteristics			
Age Distribution	n(%)	Marital Status	n(%)
21–30	16(3.8)	married	285(68)
31–40	92(22)	widow	48(11.5)
41–50	119(28.2)	single	23(5.5)
51–60	92(22)	separated/divorced	7 (1.7)
61–70	50 (12)	unspecified	57 (13.3)
71–80	32 (7.6)		
> 80	7 (1.6)	Religion	
unspecified	12 (2.8)	christian	296 (70.5)
mean: 50.6 ± 13.6 years,		muslim	113 (27)
Median: 49 (IQR 40-60 years)		unspecified	11 (2.5)
Educational Status		Tribe	
tertiary	144 (34.3)	yoruba	412 (98)
secondary	124 (29.5)	others	8.0 (2.0)
primary	66 (15.7)		
none	79 (18.8)	Place of Interview	
unspecified	7 (1.7)	southwest	279 (66%)
		northcentral	141 (34)
Premorbid Help-seeking	n(%)	Use of SBE	n(%)
self-medication	139 (33.1)	daily	25 (6.0)
visit doctor/nurse/CHEW	139 (33.1)	weekly	10 (1.0)
visit chemist/pharmacist	45 (10.7)	monthly	29 (7.0)
Alternative care	29 (6.9)	occasionally	60 (14)
observe/tell relation	6 (1.4)	Not perform	296 (72)
unspecified	62 (14.8)		
The pattern of Symptom Detection		Exposure to CBE, Mammo or USS	
Inadvertent	404 (96)	yes	16 (4)
During SBE or walk-in screening	10 (2.5)	no	404 (96)
Pain drew attention	4 (1.0		
Husband detected	2 (0.5)	Aware of Breast Cancer	
First Healthcare Provider	n(%)	yes	289 (68.8)
doctor	301 (71.7)	no	108 (25.7)
nurse	55 (13.1)	unspecified	23(5.5)
chemist/pharmacist	26 (6.2)		
breast surgeon	25 (6.0)		
CHEW	6.0 (1.4)		
others	3 (0.7)		
unspecified	4 (1.0)		

*CHEW* community health extension worker, *CBE* clinical breast examination, *Mammo* mammography, *SBE* self breast exam, *USS* ultrasound scan, *IQR* interquartile range

The Demographic characteristics of respondents showing age distribution, educational status, marital status, religion, occupation, tribe, place of the interview, the respondents’ premorbid pattern of help-seeking for medical service and the premorbid utilization of breast cancer screening modalities

### Comparative length of intervals

The PCI (median 106, 13–337) was significantly longer than the HSI (median 42, 7–150), Wilcoxon-Signed Rank test *p* = 0.0001.(paired t-test mean difference 140 ± 442 days (95% CI 95–186). Most respondents disclosed early within 30 days (330 (81, 95% CI 77–85) and consulted FHP within 60 days (230 (60, 95% CI 53–63). Most respondents had long PCI of > 30 days.(1–7 days in 91(25% (95% CI 20–29), 1–30 days in 134 (36 95% CI 31–41) and > 30 days in 237 out of 377(64 95% CI 59–68). The SCI was > 90 days in 293 of 401 (73% (95% CI 68–77), 91–180 days in 70 of 401 (17% (95% CI 14–22) and > 180 days in 226 of 401 (56% (95% CI 51–61) (Table 2).

**Table 2:** Showing the cumulative number of respondents with increasing time segments in the intervals: Most respondents disclosed early and consulted FHP early. Most respondents had a long primary-care interval and an extended detection to specialist interval

Results of time spent by respondents in the intervals									
Interval in days	1–7 (%)	1–14 (%)	1–30 (%)	1–60 (%)	1–90 (%)	Mean	Median	Range	IQR
appraisal(*n* = 407)	250 (61)	285 (70)	330 (81)	346 (85)	366 (90)	44 ± 131	6.0	1–1469	1–28
help-seeking (*n* = 397)	105 (26)	139 (35)	196 (50)	230 (58)	273 (68)	114 ± 202	42	1–2190	7–150
primary-care (*n* = 371)	91 (25)	95 (26)	134 (36)	167 (42)	190 (48)	256 ± 377	106	1–2176	13–337
detection-to-specialist (*n* = 401)	16 (4)	26 (6)	48 (12)	83 (20)	108 (26)	363 ± 409	240	1–2300	90–372

Showing the cumulative number of respondents with increasing time segments in the intervals: Showing the cumulative number of respondents with increasing time segments in the intervals: Most respondents disclosed early and consulted FHP early. Most respondents had a long primary-care interval and an extended detection to specialist interval

### Pattern of disclosure and factors influencing API

Most respondents informed the first person (primary person) early, and the husband was the most common primary person. The primary person offered the correct advice often (Table 3b), and 276 of 399 (69.2%) acted in tandem with the advice received within 2 weeks. Patronizing orthodox care, being married, and being younger were associated with early disclosure (Table 4) in the unadjusted logistic regression analysis. In the adjusted analysis combining age, premorbid preference, and marital status to predict early disclosure, only premorbid preference and marital status were significant.

**Table 3:** Showing the distribution of the first person(person1) the respondents informed about their breast symptom(s), the pattern of directives received from the first person(Person1), the first orthodox medical personnel, the number of persons informed and number of personnel visited

a. Distribution of the first person informed			
Person1 informed (*N* = 419)		n(%)	
husband		212 (50.6)	
child	Unspecified child	47 (11.1)	
	son	9 (2)	
	daughter	6 (1.4)	
sibling	Unspecified sibling	3 (0.7)	
	male	6 (1.4)	
	female	31 (7.6)	
parent	Unspecified parent	2 (0.4)	
	father	2 (0.4)	
	mother	20 (5.0)	
extended family/in-laws		6 (1.4)	
unrelated persons	friends/co-worker	15 (3.7)	
	neighbor	4 (0.8)	
doctor		24 (6.0)	
nursing personnel	CHEW	3 (0.6)	
	Certified nurse	17 (4.0)	
chemist/pharmacist		3 (0.8)	
spiritual leader		9 (2.0)	
b, Advice received from person1 or FHP			
Advice from first person	n(%)	Advice from FHP	n(%)
visit general practitioner	215 (55)	Visit doctor	17 (5.5)
Visit a breast surgeon	11 (2.9)	Visit surgeon	139 (44.8)
Visit nurse	6 (1.6)	investigate	129 (41.6)
Investigate	13 (3.2)	Antibiotics/injection/gel	5 (1.6)
Antibiotics/ gel	12 (3.0)	consult spiritual leader	3 (1.0)
chemist or pharmacist	9 (2.5)	observe/reassured	17 (5.5)
Excise	5 (1.5)		
Alternative	40 (11)		
Observe/Reassured/ tell	48 (12)		
Go hospital	24 (6.0)		
People informed(range 0–11, median: 2)		Number of FHP visited(range: 0–4, median: 1)	
0–1	152 (37)	0–1	296 (73)
2–3	170 (42)	2–3	107 (26)
> 3	86 (21)	> 3	4 (1.0)

*CHEW* community health extension worker, *FHP* First Healthcare Provider

Table showing the distribution of the first person(Primary person) the respondents informed about their breast symptom(s), the pattern of directives received from the first person(Primary person), the first orthodox medical personnel, the number of persons informed and number of personnel visited

**Table 4:** Showing the probability of short appraisal or short help-seeking interval based on the specific sociodemographic characteristics, the premorbid exposure, and experience(s) after disclosure and during help-seeking

Variable	N	Probability of short appraisal(%)	*P* value	Variable	N	Probability of short help-seeking (%)	–
Age bracket			0.02	Age bracket			0.36
20–40	144	82		20–40	112	56	
41–60	207	85		41–60	204	61	
> 60	85	71		> 60	80	52	
Education				Education			0.15
none	76	76	0.56	none	70	47	
primary	62	79		primary	63	57	
secondary	122	80		secondary	115	57	
tertiary	140	86		tertiary	136	65	
Marital status			0.03	Marital status			0.06
married	276	85		married	269	58	
single	23	65		single	22	36	
divorced/separated	57	67		divorced/separated	9	50	
widow	47	70		widow		61	
Awareness			0.36	Awareness			0.08
aware of BC	281	83		aware of BC	271	61	
unaware of BC	106	77		unaware of BC	104	57	
Premorbid health service preference			0.02	Premorbid health service preference			0.01
hospital(doc/nurse)	80	84		hospital(doc/nurse)	178	65	
self-medicate	137	82		self-medicate	131	52	
alternative	33	64		alternative	33	36	
Tumor size at detection			0.56	Tumor size at detection			0.12
1–5 cm	345	81		1–5 cm	341	57	
> 5 cm	48	81		> 5 cm	45	64	
				Person1 advice			0.02
				correct	252	63	
				incorrect	107	48	
Association with short Appraisal				Association with short Help-seeking interval			
		OR	AOR			OR	AOR
> 60		1		Incorrect advice		1	
41–60		2.3 (1.3–4.2)	1.3 (0.6–3.0)	Correct advice		1.9 (1.2–3.0)	1.7 (1.1–3.0)
≤40		2.0 (1.3–3.8)	2.0 (0.9–4.4)				
				alternative		1	
not in relationship		1		Self-medicate		1.9 (0.9–4.1)	1.5 (0.7–3.6)
married		2.6 (1.4–4.6)	2.1 (1.1–4.2)	Hospital goer		3.2 (1.5–7.1)	2.5 (1.1–5.9)
alternative		1		Association with short Primary-care interval			
self-medicates		2.6 (1.1–5.9)	2.3 (0.8–5.9)			OR	AOR
hospital goer		3.0 (1.3–6.7)	3.1 (1.2–8.1)	small tumor		1	
				big tumor		1.6 (1.0–2.4)	0.7 (0.4–1.2)
				incorrect advice		1	
				correct advice		2.1 (1.2–3.9)	2.0 (1.1–3.5)

Table showing the probability of short appraisal or short help-seeking interval based on the specific sociodemographic characteristics, the premorbid exposure, and experience(s) after disclosure and during help-seeking

### Patterns of FHP attendance and factors influencing HSI

Most respondents (355 of 417(85 95% CI 81–88) first sought orthodox medical care. The most common FHP was a general practitioner (Table 1). A total of 63 (15% (95% CI 12–19) first sought alternative care. The majority of respondents who were hospital goers before detecting their breast symptom still visited a hospital first for treatment (275 of 323 (85 95% CI 81–89). The odds of visiting hospital first vs. switching to alternative care was 2.3 (1.0–5.1) among this subgroup of patients.

Receiving correct advice(asking the patient to visit a hospital, to visit orthodox healthcare provider, or go for investigation) from person1 and patronizing hospital for other illnesses were both associated with short HSI (Table 4). There was a weak correlation between the length of API and the length of the help-seeking interval.

$$ \mathrm{r}=0.13\ \left(95\%\mathrm{CI}\ 0.03-0.23\right) $$

### Factors influencing the length of the PCI

More respondents with big (> 5 cm) tumors received correct advice compared to those with small tumors (Risk difference 5.5% (95% CI 4.0–15). The probability of correct advice was higher among the doctor FHP compared to nondoctor FHP (Risk difference 8.4 (95% CI 3.2, 20). In the unadjusted analysis, receiving correct advice and having a big tumor were associated with short PCI. Only receiving correct advice was significant in the adjusted odds ratio (AOR) (Table 4).

### Relationship between the component intervals and the SCI

The PCI strongly correlated with the SCI (r = 0.9, 95% CI 0.88–0.92). Other intervals correlated weakly with the SCI. (API r = 0.3 95% CI 0.22–0.40 and HSI r = 0.38, 95% CI 0.30–0.47).

There was a high probability of having a short SCI after traversing any component interval quickly (Table 5). The odds ratio (OR) for a short SCI vs. long SCI among those who had short API was 6.5 (95% CI 2.6–16.7), among those who had short help-seeking was 11 (95% CI 5.4–2.1) and among those who had short primary-care was 8.3 (95% CI 5.0–14).

**Table 5:** Showing the probability of short primary-care and symptom-detection to specialist interval based on specific sociodemographic risk factors, premorbid exposure and the experience(s) after disclosure and during help-seeking

Variable	N	Probability of short Primary-care(%)	P value	Variable	N	Probability of short total interval (%)	P value
Age bracket			0.11	Appraisal interval			0.001
20–40	102	29		short	314	31	
41–60	189	32		long	75	7	
> 60	79	43		Help-seeking interval			0.001
Education				short	221	41	
none	73	40	0.09	long	160	7	
primary	61	26		Primary-care interval	63		0.001
secondary	106	35		short	124	52	
tertiary	124	30		long	247	12	
Marital status			0.60				
married	254	34					
single	20	40		Incorrect advice			
divorced/separated	8	50		Doctors(GP)			
widow	45	33		Antibiotics/gel	19		
Awareness of CaB			0.16	excise	29		
aware	252	31		Observe/reassure	4		
unaware	98	37					
Premorbid health service preference			0.34	Nurse/CHEW			
hospital care(doc/nurse)	166	37		Antibiotics/gel	13		
self-medicate	122	34		excise	2		
alternative	33	24		observe	2		
Tumor size at arrival at primary-care			0.03	Chemist/pharmacist			
1–5 cm	211	29		antibiotics/gel	9		
> 5 cm	147	39		excise	1		
Personnel advice			0.02				
correct	257	37					
incorrect	76	20					
No of personnel visited			0.17				
0–1	260	36					
> 1	100	28					
Correct advice in subgroups of respondents	n	Probability of correct advice(%)					
big tumor (> 5 cm)	143	75					
small tumor (≤5 cm)	222	69					
≤40 years *n* = 104	104	78					
41-60 years *n* = 194	194	70					
> 60 years *n* = 78	78	76					
doctor FHP	292	69	0.17				
nondoctor FHP	86	40					

Table showing the probability of short primary-care and symptom-detection to specialist interval based on specific sociodemographic risk factors, premorbid exposure, and the experience(s) after disclosure and during help-seeking

Among those who divulged reasons for the long help-seeking intervals, symptom misinterpretation or symptom accumulation was 92 (47%), socioeconomic reasons were 47 (24%), and ignorance was 6 (3.0%). Reasons for long primary-care intervals was misdiagnosis by a health care provider in 37 (25%) (Table 6).

**Table 6:** shows the reasons reported for long help-seeking intervals (> 60 days) or long primary-care intervals(> 30 days) by respondents

Reason for help-seeking interval > 60 days or Primary-care interval > 30 days	Help-seeking, n-172 (%)	Primary-care, *n* = 167 (%)
Symptom misinterpretation/symptom accumulation/misdiagnosis		
ignorance	6 (3.5)	
pregnancy/lactation/menopause	8 (4.6)	1.0 (0.6)
thought benign/though will disappear	50 (29)	22 (13.0)
small size	2 (1.2)	
lump only	2 (1.2)	
no pain	19 (11)	12 (7.0)
thought boil/using antibiotics	15 (8.7)	1.0 (0.6)
thought ringworm/skin change only	1.0 (0.6))	
Navigation in primary care		1.0 (0.6)
Misdiagnosis/Investigations		46 (27.5)
Socioeconomic		
busy schedule	3.0 (1.7)	
financial constraint	18 (10.5)	33 (19.7)
family issues	2.0 (1.2)	2.0 (1.2)
distance	3.0 (1.7)	
secrecy	1.0 (0.6)	
spiritual	10 (5.8)	4.0 (2.4)
herbal care	10 (5.8)	7.0 (4.2)
Others		
reassured	7.0 (4.0)	7.0 (4.2)
strike	4.0 (2.4)	8.0 (4.7)
fear of diagnosis/panic	5.0 (2.9)	16 (9.5)
fear of mastectomy	6.0 (3.5)	6.0 (3.5)
mistrust orthodox		2.0 (1.2)

### Impact of interval length on the tumor size and risk of T-category progression

The self-reported tumor size of 13 patients among the 420 records were unreliable and excluded from the analysis of the growth in tumor size and risk of tumor size progression. Most tumors were estimated as early T-category at detection, whereas most were locally advanced at the specialist clinic (Table 7). Mean difference in T-size was significant(. paired t-test mean difference 5.0 ± 4.9 cm (95% CI 4–5), median 3.0 vs. 8.0 Wilcoxon-Signed Rank test *P* = 0.0001).

**Table 7:** Showing tumor size at various time segments in the continuum from detection to specialist clinic. Also, showing the risk of progression in tumor size per time segment

	Variables at detection			Variable at specialist clinics				
Interval Length (days)	Mean T-size (cm)	Median T-size (cm)	IQR	Mean T-size (cm)	Median T-size (cm)	T-size IQR (cm)	^#^Risk of T-stage migration (% with 95% CI)	*Risk of locally advanced disease at arrival in SC (95% CI)
1–30 *N* = 47	4.0 ± 2.0	4.0	2.0–5.0	5 ± 3	4.0	4.0–6.0	19 (8–40)	17 (6–33) *N* = 36
31–90 *N* = 58	4.0 ± 2.0	4.0	3.0–4.0	7 ± 4	6.0	4.0–8.0	54 (39–68)	46 (32–61) *N* = 50
> 90 *N* = 296	4.0 ± 2.0	3.0	2.0–4.0	9 ± 5	8.0	6.0–10.0	76 (78–89)	74 (68–80 *N* = 170
T-size	number at detection			the number at the specialist clinic				
T1	162 (40%)			32 (8%)				
T2	192 (47%)			99 (24%)				
T3	50 (13%)			278 (68%)				
Mean	3.0 ± 2.0 cm			8.0 ± 5.0 cm				

^a^risk of migration to locally advanced among respondents whose disease was early at detection (i.e., risk of migrating from T1 to T3 or from T2 to T3). # risk of migration to the next T- stage (i.e., risk of migration from T1 to T2 or from T2 to T3)

NB. The records of respondents who had tumors > 5 cm at detection were excluded from the analysis of stage migration since we would be unable to observe further stage migration according to the tumor size staging using the AJCC 7th edition

There was a moderate correlation between the length of the total interval and the growth in tumor size (r = 0.4). The average growth in the tumor size per month was estimated to be 0.4 cm in the first 12 months. The risk of tumor progression within the first 12 months was lowest in the first month (Table 7). The overall risk that a lump would be locally advanced when detected inadvertently was 12% (95%CI 9–16), and the risk that it would migrate to the next T-category before arriving in a specialist clinic was 64% (95% CI 59–69). The OR for T-category progression in SC interval of 31–90 days vs. 1–30 days was 5 (95% CI 2.0–12), and the OR in SC interval > 90 days vs. 1–30 days was 16 (95% CI 7.0–38). Among patients who detected their tumors relatively early (estimated as T1 or T2), the hazard of progressing to advanced-stage increased with time. The hazard was lowest in the first 30 days (3%), 17% in 60 days, 31% in 90 days and 61% in 180 days.

## Discussion

In this survey, two-thirds of the respondents stayed longer than three months between detecting BC symptoms and arriving in a specialist clinic. The PCI was the longest interval, and there was a strong correlation between the length of the PCI and the SCI. Symptom misinterpretation and misdiagnosis were frequent reasons for extended intervals. The majority of the patient detected their lesion early, but the majority were already locally advanced before arriving in a specialist clinic.

At least two-thirds of our respondents first visited orthodox personnel to seek help and, a similar proportion consulted FHP early in tandem with their advisor’s directives. We did not establish the direct influence of advisors on the women’s decision nonetheless, the association is consistent with the report in South Africa [16], where patients acted based on pressure from relations. The husbands were the most frequent advisors; hence, they are a potential focus for intervention. Engaging men to promote uptake of positive breast health activities is useful in places where women rely on husband and family support [20], notably, in Africa, where the men dominate the leadership role [21] and politics.

Similar to previous reports in Nigeria, [9, 22] a large number of our respondents preferred orthodox care, and the pattern of help-seeking was consistent with their premorbid preference for health care services. This indicates that without unfavorable experience(s), it is unlikely that women will suddenly change their health care preferences once they detected their breast lesions. We can exploit the premorbid conditioning by improving access to our hospitals. Raising satisfaction derived in the hospital for treatment of other minor illnesses might build confidence and enduring relationship between potential breast cancer patients and the clinicians.

The longer PCI and its dominating influence compared to the other intervals supports some reports and negates others. Harirchi in Tehran [23] and Yau et al. in Hong Kong [24] reported a higher proportion of patients with help-seeking delay. Moodley et al. first reported a higher proportion of delay in the patient interval among 20 patients in South Africa [16] and then in a subsequent study of 201 patients; they reported a higher proportion of long delays in the system’s interval [25]. Roy et al. [26] in Bangladesh and Maghous et al. [27] in Morocco, both reported that doctors were complicit in a third of long interval situations [26, 27]. Also, in Nigeria, Ezeome et al. [9], Ayoade et al. [17] and Akinkuolie et al. [20] reported disease progression during the primary-care interval.

The most frequent reason for a long primary-care interval in this study was misdiagnosis by the FHP. We found that smaller tumors were associated with longer intervals. We suspect that smaller tumors were more challenging to evaluate because of limited symptomatology. Instances of symptom misinterpretation and misdiagnosis were also prominent reasons for extended intervals in other studies in Nigeria [4, 28], other parts of Africa [16, 27, 29–31], middle east [32] and Asia [28], Ensuring triple assessment rather than depend on physical finding to initiate treatment may reduce misdiagnosis. In our study, the risk of incorrect advice was higher among nondoctor FHP compared to doctor FHP. The doctor FHP and nondoctor FHP had different error patterns, which should be noted during education campaigns.

We found that there is an increasing probability of transitioning from early to locally advanced disease as time elapsed, and the risk of transitioning was least in the first 30 days after the detection of early disease, and it more than doubled afterward. One out of every ten women who detected their lumps inadvertently were already locally advanced. Furthermore, one out of every three was likely to be advanced among those who arrived in a specialist clinic after 30 days. This suggests that the strategy to promote early detection and treatment of clinically symptomatic BC in low resource settings [33, 34] may be effective in our patients if implemented with a tight timeline. Although we could not assess the influence of tumor biology on disease progression in this study and we assumed that time was an independent predictor of tumor progression, the common timeline in our literature describing detection to presentation of more than three months as late [9, 17, 27] was lax for this cohort of respondents because at least a third already experienced significant tumor growth within 90 days.

The clinical implication of for long detection to treatment interval is not adequately researched in Africa. In a population of BC patients in southern Africa [18], more than 20% were locally advanced in a median time to treatment of 110 days. In Ghana [35], patients who stayed a total interval shorter than 2 months had smaller tumors compared to the total interval of 12 months. In contrast, two-thirds of patients who stayed longer than six months in a study in Uganda [36] still had an early disease. We need more studies to describe the relationship between total interval and outcome in Africans.

Our study is the first to explicitly show the relationship between premorbid experience and the pattern of help-seeking among breast cancer patients in sub-Saharan Africa. Our study is also the first to show the likely changes in breast tumor size as time elapsed in a cohort of breast cancer patients in sub-Saharan Africa and the first to show the relationship between the component intervals and their relative influence on the time to a specialist clinic.

This research is limited in that the primary outcome was patient-reported; hence it might be influenced by recall bias. We attempted to minimize the bias by interviewing the patients within four weeks of arriving in the specialist clinic. Moreover, we helped them to cast their minds back on significant events occurring around the recalled dates or periods.

The self-reported tumor size based on patients’ retrospective recall may be inaccurate, and we could not triangulate for its accuracy by comparing the respondent’s recall with the primary-care records due to poor record-keeping. Also, we did not evaluate the interaction between tumor biology, the elapsed time, and tumor progression. We were unable to find other ways of estimating tumor size at detection because it was a prehospital event, and we were unable to find other ways of estimating tumor size at contact with the FHP. Nonetheless, we attempted to minimize inconsistency in the self-reported tumor size by using the estimate given by the patient at all points for the analysis, and we excluded overtly inaccurate estimates.

## Conclusion

Most patients in this study visited the FHP early; however, most stayed longer than 3 months between symptom detection and arriving in a specialist clinic with significant tumor progression in the interval. The PCI was the longest interval. The most common reasons for long intervals were symptom misinterpretation and system-related factors.

## Supplementary information

**Additional file 1.** Data collection questionnaire.

## Data Availability

Data limited for interpreation of results for this research is available on reasonable request to the corresponding author and as supplementary file.

## Abbreviations

API: Appraisal interval
AOR: Adjustted odds ratio
AJCC: American Joint Committtee on Cancer
BC: Breast cancer
BSE: Breast self-Examination
CBE: Clinical breast examination
CI: Confidence interval
CHEW: Community health extension worker
FHP: First healthcare provider
HIS: Help-seeking interval
IQR: Interquartile range
LMICs: Low and middle income countries
Mammo: Mammography
NS: Not significant
OR: Odds ratio
PCI: Primary care interval
r: Correlation coefficient
SCI: Symptom detection to specialist interal
T: Tumor size
