# Supplementary material for: Presentation intervals and the impact of delay on breast cancer progression in a black African population
Source: BMC Public Health. 2020 Jun 19;20:962. doi: 10.1186/s12889-020-09074-w (PMC7304119; doi:10.1186/s12889-020-09074-w)
Supplement: Supplementary file 1 — Additional file 1. Data collection questionnaire. [file 12889_2020_9074_MOESM1_ESM.pdf]

Finding the major drivers of continuing late presentation of breast cancer in Southwestern and Northcentral Nigeria

COVER LETTER AND CONSENT

Dear Respondent,

Good day, and thank you for your time. We want to seek your consent to ask questions about the lump in your breast over the next 20 to 30 minutes.

Let us to start by telling you our purpose

The purpose is to conduct a research about women's treatment journey when they notice lump(s) in their breast. This research will assist us tremendously to understand how to improve our care of women who have the kind of lump in your breast.

The research is approved by the hospital and is being conducted by doctors who treat these kind of breast lumps in this hospital.

I am {*the recruiter's name: an investigator*}.

I am one of the investigators in this research, with your consent I will invite someone who will be asking you questions and they will record your response. Every response you give will be confidential.

You can choose to respond or not respond as you wish.

If you like, you can choose to stop the session at any time, but as earlier state this should take about 20-30minutes.

If you have any questions, you can ask me or your interviewer at any time in the course of this session and if you have any questions at the end of this session you can call any of these 2 numbers:

Local leads' number:

Primary investigators' number: +2348068756812

Please note that partaking in this question and answer session is not mandatory and will not affect your treatment in any way.

If you are willing to partake in this session, please sign/thumb print here before we proceed

Respondent's Name	Signature/thumbprint

PERSONAL INFORMATION PAGE(to be

SN

============SECTION TO BE COMPLETED BY LEAD INVESTIGATORS=======================

SITE: [… ….]

Respondent's Surname (record in numbers refer to codebook)

Respondent's GivenName (record in numbers refer to codebook)

Age (years) (Record in alphabets refer to codebook) HOSP No (Record in alphabets refer to code book)

Phone number (Record in alphabets refer to codebook) **Education level** [none] [pri] [sec] [tert]

Home Address:

Tribe: Occupation

Marital statu

===========STARTING POINT FOR INTERVIEWER RECORDING=================

(**Start interview by reading out the coded name, coded hospital number and serial no**)

Date first visit to study center: dd / mm / yyyy

first visit to specialist clinic: dd / mm / yyyy

Date of referral to specialist center: dd mm yyyy

Date of interview dd / mm / yyyy

All fields are required. If not applicable please state **"not applicable"**

All fields are required. If not applicable please state **"not applicable"**

1 What first drew your attention to noticing this lump in your breast? (*context creating aiding recall*)

2 How long has it been since you first noticed it. ☐ days ☐ weeks ☐ months ☐ years

3 Use your finger(s) or fist to show me how big it was when it was first noticed.

Describe patients' response here and estimate the size in cm

4 Has it changed in size since the first time it was noticed? ☐ YES ☐ NO

5 If yes to (4) Please show me how big it is now using your finger(s) or your fist

Describe patients' response here and estimate the size in cm

6 Who are the people you that you discussed this lump with?: [tick all that apply] Mother/ father/ female siblings/ male siblings/ friends /neighbor / Husband / co-workers / doctor / Nurse / pharmacist/ chemist / CHEW /spiritual leaders / herbalist / others specify

7 Please re-trace the order in which these people in (6) were informed(the first person is Y, no names just relationships)

____

____

____

8 How frequently do you perform SBE. [not performed] [daily] [weekly] [monthly] [occasionally]

8b Have you had CBE or USS or Mammo in the past ? Yes / No (circle CBE, USS or Mammo as apprpriate)

9 What was the interval between noticing the lump and telling the first person Y: ☐ ☐ ☐ ☐

days weeks months years

10 What was the first person(Y)'s advice/directive:

____

____

11 What was the advice or directive given by the others (specify person and directive)

____

____

____

12 What do you first do when you have health related problems or conditions such as fever, malaria etc

SN

All fields are required. If not applicable please state **"not applicable"**

13 List the facilities and health persons you have visited to receive treatment or advice for this lump since you noticed it (e.g. hospital /chemist /pharmacy /Nurse /doctor)(for hospital specify: private, primary center, secondary center tertiary center):

____

14 Specify the first health personnel(X) you visited to receive treatment or advice: eg chemist, pharmacist, family doctor, specialist, CHEW (**no names just cadre of personnel or facility**) [ ]

15 What was the interval between noticing the lump and visiting personnel X: [ ] days [ ] weeks [ ] months [ ] years

16 In the re-tracing in (7)above what is the position of this health personnel X [ ]

17 Use your finger or fist to show me how big the lump was when you first visited personnel X:

Describe patients' response here and estimate the size in cm

18 What was the personnel X's advice or directive?

____

____ 19

What was the directive of other health persons(in 13 above)?

____

____

20 How long ago did you visit a pastor about this lump in your breast? [ ] days [ ] weeks [ ] months [ ] years

If yes, what is the position of the pastor along the re-tracing above [ ]

21 What was the pastor's advice/ directive?

____

22 How long ago did you visit an Islamic cleric about this lump in your breast? [ ] days [ ] weeks [ ] months [ ] years

If yes, what is the position of the Islamic cleric along the re-tracing above? [ ]

23 What was the Islamic cleric's advice/directive?

____

24 How long ago did you visit a native healer about this lump in you breast? [ ] days [ ] weeks [ ] months [ ] years

If yes what is the position of the native healer along the re-tracing above? [ ]

25 What was the native healer's advice/directive?

____

26 What is the native healer's title (*what people call him, how is he referred in your native language or dialect*)

[ ]

All fields are required. If not applicable please state **"not applicable"**

SN

29 From whom or where did you first hear/learn about breast lumps?

30 When, how long ago did you first hear about breast lumps days weeks months years 31 Breast lumps

____

____

{if don't know types, divulge that there are ordinary and cancerous types}

32 What treatment is given to ordinary breast lumps in the hospital?

____ 33 What

hospital?

____

34 When/how did you first learn about the treatment given to cancerous breast lumps in the hospital

How? When? day weeks months years

35 When/how did you first learn about the treatment given to ordinary breast lumps in the hospital

How? When ? days

36 This lump in your breast, what was it called in the hospital (or what type was it called in the hospital)?

____

37 What other names have people who are not medical personnel called this lump in your breast (pastor, Islamic cleric and r

____

____

38 Do you know/have you heard (*circle know or heard or both*) about someone treated for ordinary breast lumps in the hospital before the lump in your breast was noticed ? YES NO

39 Where is the fellow now?

____

40 Do you know/have you heard (*circle know or heard or both*) about someone treated for cancerous breast lumps i hospital before the lump in your breast was noticed? YES NO

41 Where is the fellow now?

____

42 What options have you been offered by the various health personnel you have visited as treatment for this lump in your b

____

____

43 What is your religion? Christianity / Islam / others specify:

***All fields are required. If not applicable please state* "not applicable"**

44 How far is your home from the referring hospitals? Quantify in hours' drive: [ ] hours' drive :

____

45 Why did you decide to come here ? (referred, not getting better, advice by friends etc)

46. How far is your home from here ? quantify in hours' drive [ ] hours'

drive 47 What type of biopsy has been done? none / FNAC / Open biopsy

48 When was the biopsy done?
49 Where was the biopsy done?

50 What is the result of the biopsy : specify:_________________________/specimen not sent for testing / don't know

51 if interval between noticing lump and visiting orthodox care is > 8 weeks ask why. Insert reason here ________
_____________________-____-
52 if interval between first orthodox visit and visiting doctor is >2 weeks ask why. Insert reason here ____________
____

53 if interval between visiting first doctor and visiting specialist is >4weeks ask why. Insert reason here____

54 What is your pressing concern about hospital treatment offered to you for this lump (stressful, expensive, mutilating,
Personnel attitude etc)____

54. who to you first visit if you have to consult a health personnel for medical treatment__________________

55. If the place first visited for the care of her breast lump is different from the place usually visited for other illnesses in question 54 above, find out why____

56. If patient visited alternative care after first visiting hospital ask why____

57. If delayed or absconded after visiting specialist ask why. Insert reason here__________________________________

____

(final action : the local lead reviews questionnaire for completeness) Thank you very much, we are done.

58. **Translator required during interview** [YES] [NO]

------------------------**Lump information and signing off *Section to be completed by local lead investigator***---------------------

Background information from patients' case note.

Source of referral: specify: [ ]

Diagnosis: [ ] Tumor size (cm) [ ]

Name of investigator [ ] Signature [ ]
